# Risk factors and prevalence of use of different tobacco products among school adolescents in the North Central region of Morocco: a cross-sectional study

**DOI:** 10.11604/pamj.2018.30.73.10896

**Published:** 2018-05-29

**Authors:** Belkacem Bendaou, Btissame Zarrouq, Khaoula El Kinany, Badiaa Lyoussi, Mohamed Chakib Benjelloun, Chakib Nejjari, Karima El Rhazi

**Affiliations:** 1 Laboratory of Epidemiology, Clinical Research and Health Community, Faculty of Medicine and Pharmacy, Sidi Mohammed Ben Abdallah University, Fez, Morocco; 2 Laboratory of Physiology-Pharmacology and Environmental Health, Dhar El Mahraz Faculty of Sciences, Sidi Mohammed Ben Abdallah University, Fez, Morocco; 3 Pneumology Service, Centre Hospitalier Universitaire Hassan II of Fez, University Sidi Mohamed Ben Abdellah, Fez, Morocco

**Keywords:** Tobacco, adolescence, school students, prevalence, risk factors, Morocco

## Abstract

**Introduction:**

The rising number of youth smokers is a major concern to public health in Morocco. The implementation of appropriate preventive measures would require information about the prevalence and determinants of tobacco use. Data on tobacco consumption among adolescents in the North Center of Morocco are scarce. Therefore, the current study aims at investigating the risk factors of smoking and the prevalence of the use of different forms of tobacco among school teenagers in the North-Centre region of Morocco.

**Methods:**

A cross-sectional study was conducted in North Central Region of Morocco among students in public secondary schools selected by stratified cluster random sampling. The statistical unit devised was a school class from the seventh to the twelfth grade of the Moroccan educational system.

**Results:**

A total of 3020 students (53% were males) and an average age = 16 ± 2.1 years were included in the study. The prevalence of the use of cigarettes was 16.1% (95% CI: 14.8% - 17.4%).For other tobacco types such as hookah, snuff and tobacco chewing, the prevalence was respectively 70.6%, 42.8% and 35.0% for cigarettes smokers. The level of current tobacco use was noticeably higher among boys (15.0% vs. 2.5%, p < 0,001) and high school students in comparison to middle school pupils (21.2% versus 11.9%; p < 0,001).

**Conclusion:**

Among young people, all types of smoking are growing increasingly in our Moroccan society; this alarming result can contribute to help the decision-makers to make decisions and force us obviously to take preventive measures rapidly.

## Introduction

Teenage smokers are a major concern to public health worldwide [1-3]. World Health Organization (WHO) reports 4.9 million deaths among adolescent smokers, and the number is likely to go beyond 8.4 million by 2020. Currently, 70% of the deaths are registered in developing countries [4,5]. As it is known, smoking is introduced most notably during adolescence, a phase when youngsters tend to try out almost every new thing [6,7]. It is equally undeniable that changing the bad habits left by adolescence, shifting to adult life is a hard thing to do. Therefore, early smokers are prone to addiction later on in their lives [8]. In Morocco, Smoking Moroccan Study (MARTA), an epidemiological study reveals that adults generally take up smoking at the age of seventeen whereas youth below the age of nineteen try their first cigarette at their fourteenth year on average [9]. According to the Global School Health Survey (*GSHS*), conducted in 2010, the prevalence of student smokers within the last 30 days reached 5.2% [10]. By the same token, the Global Youth Tobacco Survey (GYTS) carried out in Morocco in 1999 by WHO, Disease Control and Prevention Center (CDC), and the Canadian Association for Public Health (L’ACSP) reported that 9.5% of teenagers use different forms of tobacco [11]. The Mediterranean school project on alcohol and drugs (MedSpad, 2013),which was conducted in 2013, revealed that 16.4% of Moroccan school students used hookah, 12.4% used snuff tobacco and 9.1% used chewing tobacco [12]. Risk factors of smoking among adolescents are numerous. They include demographic socio-economic, behavioral, and environmental elements. Males, poorly educated or uneducated parents [13,14] , single or divorced parents are more liable to the predominance of the phenomenon [2,15]. In terms of behavior, low school achievement, risk-taking tendency, lower self-esteem and weakness of character are major causes of smoking. Other reasons trigger the addiction. Home terms of conduct are not far-fetched. If a teenager has parents, a brother or a friend who smokes, he/she will more eagerly take up smoking by imitation [13,16,17]. Regarding the studies conducted on the national scale we can easily notice some insufficiency at a regional level. To the best of our knowledge, it’s first of its kind in the North Central region of Morocco targets to identify the risk factors of smoking and the prevalence of the use of different forms of tobacco among school teenagers in the North-Centre region of Morocco, the cross sectional design and the large sample size allows us to collect data concerning smoking behaviours.

## Methods

### Research design

This cross-sectional study was conducted from April 2012 to November 2013. The project was approved by the Ethics Committee of the Faculty of Medicine and pharmacy of Sidi Mohammed Ben Abdellah University. The participants were students from middle and high public schools in the North Central Region of Morocco (namely regions of Fez, Boulemane, Taza, Taounate, and El Hoceima). Based on the lists of schools in this region, which were obtained from the Statistical Office of National Education Ministry, the schools were split into seven geographical regions, and classified into two strata: urban and rural. The stratified random sampling was carried out according to the type of schools (middle or high school) using the proportional allocation technique, that is to divide the sample in each stratum (urban and rural) based on the proportionality of middle and high schools depending on the total number of schools. Eventually, 44 middle schools and 24 high schools were randomly selected. Indeed, all the students from the selected classes were included in the study. The questionnaires (Additional file 1) were administered upon receiving school-headmasters’ permission and verbal consent from all participants. Concerning the students under the age of 16, verbal informed consent from the parents was also obtained. The process of contacting parents was facilitated through parents’ associations. The investigators distributed the questionnaires in the classrooms during a regular class period. The questionnaires’ instructions stated that participation was voluntary and that responses were anonymous and confidential.

### Assessment

#### Socio-demographic characteristics

A questionnaire was used to collect demographic information such as age, gender, educational level, family income, parents’ marital status and their educational level.

#### Consumption of cigarettes

We have made questions about the use of the cigarette, the number of cigarettes smoked per day, the age of first cigarette, and people who consume the cigarette in the environment. Smoking habit was defined according to the International Union against Tuberculosis and Lung Diseases guide [18]. Respondents were defined as current smokers (daily and occasional smokers) if they were smoking at the time of the survey and smoked more than 100 cigarettes in their lifetime; they were defined as ex-smokers if they smoked more than 100 cigarettes in their lifetime, but stopped smoking during more than 3 months at the time of the survey; and they were defined as never smokers if they have never smoked or smoked less than 100 cigarettes in their lifetime [18].

#### Consumption of different types of tobacco

During this phase, we have assembled data on the frequency of use of different forms of tobacco such as hookah, snuff and chewing tobacco (smokeless tobacco).

#### Statistical analysis

The data entry stage started immediately after data collection. Data were entered into MS Windows Excel in the form of codes, and transferred to the Statistical Package for Social Sciences (SPSS) software version 20 (SPSS Inc., Chicago, IL, USA). Data analysis contained descriptive and multivariate statistics binary logistic regression. A simple descriptive analysis was done for the variables of interest. The qualitative variables will be described in the form of proportions and the quantitative variables as an average. Odds ratios and Adjusted Odds ratios, along with 95% confidence intervals, were calculated. Differences in proportions were assessed by the Chi-square test. P values of < 0.05 were considered as statistically significant.

#### Abbreviations

CDC: Disease Control and Prevention Center; GSHS: Global school health survey; GYTS: Global youth tobacco Survey; MedSpad; Mediterranean School Survey Project on Alcohol and Other Drugs; WHO: World Health Organization.

#### Funding

Sincere thanks go to Hassan the 2^nd^ University Hospital Center, Faculty of Medicine and Pharmacy of Fez, University Sidi Mohammed Ben Abdallah for funding this study.

#### Availability of data and materials

Data will not be shared, because there are other findings expected to be exploited in other publications.

#### Ethics approval and consent to participate

Ethical clearance was acquired from the ethical committee of the Faculty of Medicine and Pharmacy in Fez city. The questionnaires were administered upon receiving school-headmasters’ permission and written informed consent from all participants. Concerning the students under the age of 16, written informed consents from the parents were also obtained. The process of contacting parents was facilitated through parents’ associations.

## Results

### Socio-demographic characteristics

Out of 3170 students who participated in the study, 3020 completed the questionnaires (response rate = 95.2%). There were more males (53.0%) than females (47.0%), and the average age was 16 years (SD = 2.1 years). The rest of socio-demographic characteristics is illustrated in the followingTable 1.

**Table 1:** Socio-demographic characteristics of the study population (n=3020)

Socio-demographic characteristics		Number (N)	Percentage (%)
Gender	Boys	1602	53.0
	Girls	1418	47.0
Age Range (years)	11 - 14	795	26.4
	15 - 18	1857	61.7
	19 – 23	357	11.9
School levels	Middle school	1657	54.9
	High school	1363	45.1
Parents’ marital status	Married	2684	89.3
	Divorced	109	3.7
	Widowed	145	4.9
	Separated	65	2.1
Socio-economic status	≤ 300 MAD	498	16.8
	301 – 1000MAD	2280	76.9
	> 1000 MAD	187	6.3
Residence	Urban	2273	75.3
	Rural	774	24.5
Father’s education	Illiterate	1065	35.9
	Primary school	806	27.2
	Secondary	767	25.9
	University	326	11.0
Father’s Employed	Employed	2490	88.4
	unemployed	82	2.9
	Retired	245	8.7
Mother’s education	Illiterate	1813	60.6
	Primary school	514	17.2
	Secondary	517	17.3
	University	149	5.0
Mother’s Employed	Employed	281	9.5
	unemployed	2688	90.5

### Cigarette use

The total prevalence of the use of cigarettes was 16.1% (95% CI: 14.8% - 17.4%). The Male students were more likely to smoke currently than females (15.0% vs. 2.5%, p < 0.001). High school students also had a prevalence higher than college students, (21.2% versus 11.9%; p < 0.001). The geographical factor proved decisive; in Taza Alhoceima Taounate, the rate was even higher than the one in Fez-Boulmane. It was (18.5% versus 14.4%; p < 0.001). The average number of smoked cigarettes ranged from 6.4 ± 6.1. And the mean age at which the school students started smoking cigarettes was 13.9 years (SD ± 6.5 years).

### Prevalence of various forms of tobacco consumption among cigarette smokers

The majority of cigarette smokers used other forms of tobacco. Males smokers of cigarettes were more likely than females to use hookah (70.7% versus 69.5%), snuff tobacco (45.3% Vs 21.7%), and chewing tobacco (36.4 Vs 21.7%) (Table 2).

**Table 2:** Prevalence of use of various forms of tobacco among cigarette users by gender

	Gender	Prevalence %
Hookah	Boys	70.7
	Girls	69.5
Snuff	Boys	45.3
	Girls	21.7
Chewing tobacco	Boys	36.4
	Girls	21.7

### Smoking risk factors

The invariant analysis demonstrated that males were more likely to use tobacco compared to girls (p < 0.001). As for age, it was noted that the older a person grows, the more likely she/he is to use cigarettes (p < 0.001). By the same pattern, youngsters in urban setting had higher probabilities to become tobacco users compared to their peers in rural areas (12.1% Vs 4.0%). High school students also generally smoke more than middle school students (59.5% Vs 6.5%). Adolescents with household problems (socio-psychological issues) are inclined to smoke tobacco in response compared to psychically more stable youth (13.3% Vs 2.6%). As parents education levels raise, the risk of adolescents taking up cigarettes drops, and vice versa. However, middle class children are found to smoke more than those from lower and higher social classes (Table 3). The multi-variate logistic regression analysis identified the following risk factors: age 19 – 23 years, (OR 10.2; 95% CI: 6.6% – 15.7%), male gender, (OR 7.5; 95% CI: 5.6% – 10.0%), household psychological instability, (OR 2.1; 95% CI: 1.5% – 2.9%), and father’s education level (OR 1.5; 95% CI: 1.1% – 2.2%) (Table 4).

**Table 3:** The univariate analysis of tobacco consumption among school students in the North Central region of Morocco

Variables		Smoking Prevalence %	p-value
Age range (years)	11 - 14	1.1	< 0.001
	15 - 18	11.0	
	19 - 23	3.9	
Gender	Boys	15.0	< 0.001
	Girls	2.5	
Residence	Urban	12.1	0.5
	Rural	4.0	
School levels	Middle school	6.5	< 0.001
	High school	9.5	
Socio-psychological stability	Yes	2.6	< 0.001
	No	13.3	
Father’s education	Illiterate	4.9	0.04
	Primary school	4.8	
	Secondary	4.5	
	University	2.0	
Mother’s education	Illiterate	9.6	0.02
	Primary school	2.3	
	Secondary	2.9	
	University	1.2	
Socio-economic status	≤ 3000 MAD	3.3	0.02
	3010 – 10000 MAD	11.5	
	≥10000 MAD	1.2	

**Table 4:** Multi-variatelogistic regressionanalysis of tobacco consumption among adolescents in the North Central region of Morocco

Variables		OR	95% CI	p-value
Age	11 – 14	1		0.000
	15 - 18	5.3	3.6 – 7.8	
	19 - 23	10.2	6.6 – 15.7	
Gender	Girls	1		0.000
	Boys	7.5	5.6–10.0	
psychological stability	Yes	1		0.000
	No	2.1	1.5–2.9	
Father’s education level	Illiterate	1		0.023
	Primary school	1.4	1.1 – 1.9	
	Secondary	1.4	1.1 – 1.9	
	University	1.5	1.1 – 2.2	

## Discussion

### Prevalence of cigarettes use

The present study is the first large survey focusing on different tobacco products use among middle and high school students in the North Center of Morocco. The lifetime smoking prevalence among all school students (16.1 %) reported in this study is slightly lower than the rate (17.0%) reported by Mediterranean School Survey Project on Alcohol and Other Drugs conducted in 2013 [19]. In this study the prevalence of smoking among students aged 13 to 15 years was 4%. This rate is slightly higher than the prevalence (3%) found in the report Global School-Based Health Survey (GSHS) conducted in Morocco among 2670 students in 2006 [20]; and is lower than the prevalence reported in GSHS Tunisia (7.6%) which was conducted in 2002 and targeted the same category (students aged 13-15 years old) [21]. Compared to the results generated by the Regional Office of Eastern-Mediterranean Countries in 2010, Morocco has the lowest rates of tobacco use in the Maghreb. Other countries from the MENA region are subject to higher smoking prevalence such as Tunisia with 18.6% and Syria with 19.9% [11].

### Prevalence of other forms of tobacco use

In the present study, hookah smokers represented 5.1%. This prevalence is almost the same (4.9%) found in the MedSpad Morocco in 2010 [11]. Drawing a comparison between our results and the results found by MedSpad in the Maghreb and the countries of the East-Mediterranean like Syria; the research underlines a high Hookah intake rates in Syria, Egypt and Tunisia. The prevalence rates were respectively 19.7%, 7.5% [22] and 5.8% [23]. Among cigarettes smokers, the prevalence of use of other forms of tobacco was remarkable: hookah users rise from 16.4% to 70.5%, snuff takers, from 12.4% to 42%, and tobacco “chewers”, from 9.1% to 34% [19]. This high rate appear “normal” because the majority of cigarettes smokers use other types of tobacco. The rise in tobacco intake particularly the hookah is partly due to the growing number of hookah lounges which are attracting more and more youngsters.

### Risk factors relative to tobacco use

Analyzing the data by logistical regression, we can identify a number of socio-economic risk factors behind tobacco use like age, gender, household psychological state, and parents’ education level. In fact, age remained largely a decisive element. The most affected age group by tobacco use was the 15 - 18 years old. In addition, it has been noticed that boys tend to take up smoking more precociously in comparison to girls. On the other hand, boys smoke seven times more than girls. The percentage roughly matches the figures reported by GYTS in 2000 - 2007 in Algeria where 28% of boys smoked cigarettes comparing to 2.8% of girls [24], in Tunisia (30.6% versus 8.1%) [23] and in Egypt (29% versus 7%) [22]. Contrary to some European countries, which have generally higher prevalence of female smokers and lower prevalence of male smokers [25]. In fact, the reason why female smokers represent a minority among tobacco users is that smoking among girls is still seen as shameful and inappropriate in Moroccan culture. In Moroccan society, having a cigarette between a woman’s lips certainly tarnishes her image and reputation in society [26]. In addition, smoking is also mostly coupled with family issues and parents low literacy levels, a condition where mothers and fathers are least aware of the symbolic impact of a smoker community on their children [27]. For these reasons, parental care remains one key to protect teenagers from smoking.

### Limits of the study

We note some limitations to this study. First, tobacco use is self-reported, which could be subject to social desirability bias. Since tobacco use among adolescents is a taboo practice in the Moroccan conservative society. There is a risk of underreporting among school students generally and females particularly when using self-report questionnaires. Although we went to great lengths to ensure confidentiality and anonymity, we could not assess underreporting of tobacco use. The prevalence reported above may thus represent low estimates of the actual prevalence. Second, the study is cross-sectional, which limits the extent to which conclusions can be drawn about the causal nature of the associations between the correlates and tobacco use.

### The strengths of study

First of its kind in the North Central region of Morocco, the present study focused on tobacco use among a representative sample of school students. It has managed to gauge the spread of smoking in seven Moroccan cities.

## Conclusion

Our study supports that smoking is a real threat to Moroccan youngsters now. Individual intervention is not enough and largely ineffective in the battle against tobacco. These results can be used to develop decision policies more adapted to our Moroccan context. One such a great initiative is “Tobacco-free schools” launched by Lalla Salma Foundation, treatment and prevention of cancers. More is required in this regard. For instance, policy-makers can introduce prevention campaigns and sensitize primary school pupils to the dangers of tobacco through modern technological means such as audiovisual media, advertisement leaflets, didactic tools, etc. Monitoring tobacco use among Moroccan school students is essential to study risky behaviors among adolescents and provide a pointer to potential future trends.

### What is known about this topic

- To our knowledge the number of youth smokers is higher in some regions of Morocco;
- However, the data of tobacco consumption among adolescents in the North Center of Morocco are scarce.

### What this study adds

- This is the first large study that includes seven cities in Morocco, which represent the center of culture and distribution of tobacco; therefore, this region is considered as the most exposed region to tobacco;
- The prevalence of tobacco consumption is expected to be higher than the other regions of Morocco: this assumption was confirmed by the higher prevalence compared to other cities;
- These results highlight the importance to take specific actions in collaboration with the Ministry of National Education and Public Health to sensitize adolescents in primary and secondary schools who are in a critical age and more prone to be influenced; others specifics measures have to be taken with regard to the tobacco culture limitation and/or it’s fighting.
